# The lived experience of active surveillance for prostate cancer: a systematic review and meta-synthesis

**DOI:** 10.1007/s11764-025-01748-x

**Published:** 2025-02-12

**Authors:** Russell J. Briggs, Jeff Dunn, Suzanne K. Chambers, Samantha Jakimowicz, Anna Green, Nicole Heneka

**Affiliations:** 1 Centre for Health Research, University of Southern Queensland, Brisbane, Australia; 2 Prostate Cancer Foundation of Australia, Sydney, Australia; 3 Australian Catholic University, Sydney, Australia; 4 Charles Sturt University, Bathurst, Australia

**Keywords:** Prostate cancer, Survivorship, Active Surveillance, Psycho‐oncology, Unmet needs

## Abstract

**Purpose:**

Prostate cancer is one of the most prevalent cancers worldwide. Active surveillance is a widely accepted treatment option for some localised prostate cancers. However, concerns have been raised about the experiences of men on this treatment given that almost 40% will discontinue without clinical indications. The objective of this review was to identify the lived experience of men on active surveillance.

**Methods:**

A systematic review and meta-synthesis, according to PRISMA guidelines. Studies were included if they reported qualitative data exploring the experiences of men undertaking active surveillance. Thomas and Harden’s approach was undertaken for data synthesis.

**Results:**

Five databases were searched identifying 3226 articles, and 13 studies met the inclusion criteria. Two overarching analytical themes were identified: (i) men on active surveillance live with a lack of certainty; and (ii) re-establishing agency drives resilience and facilitates confidence in active surveillance. Lack of certainty on active surveillance is derived from men feeling a loss of control over their health and/or lives. This induces a stress response of ongoing worry and anxiety and loss of agency, further driving the stress cycle. Re-establishing agency alleviates the stress response, promotes resilience, and facilitates confidence in active surveillance.

**Conclusions:**

The experience of active surveillance is underpinned by ongoing lack of certainty diminishing agency and driving cyclical stress.

**Implications for Cancer Survivors:**

It is essential that health professionals better support men to establish and maintain confidence in active surveillance. Further research into men’s perspectives of interventions and strategies that best facilitate agency and effectively support them is warranted.

**Supplementary Information:**

The online version contains supplementary material available at 10.1007/s11764-025-01748-x.

## Background

Prostate cancer is one of the most prevalent cancers to impact men, with almost 1.5 million men diagnosed in 2022 [1]. Globally, cancer is estimated to cost $25.2 trillion in international dollars between 2020 and 2050 [2]; prostate cancer being the 4th most prevalent cancer worldwide is a significant contributor to these costs [1]. Prostate cancer treatment has a significant physical side effect burden, which can often be long term and drives psychological distress [3]. Men receiving a diagnosis of prostate cancer often have high unmet psychosocial and supportive care needs [4], and up to a 70% increased risk of suicide, regardless of clinical stage or risk category at diagnosis, compared to men in the general population [5]. Men with prostate cancer are also three times more likely to experience anxiety and twice as likely to experience depression than those without a diagnosis [4, 6]. Critically, prostate cancer–related distress can be significant within the first year following diagnosis [7, 8], and the risk of suicide is greatest within this period [7]. In addition to the psychological impact of a prostate cancer diagnosis, men also face the burden of physical side effects and symptoms as a result of treatment [9]. Sexual dysfunction, urinary incontinence, and bowel disturbance are common and often long-lasting side effects of surgery and/or radiotherapy treatment for prostate cancer [10, 11].

Over the past 15 years, there have been changes to how low- and intermediate-risk prostate cancer is managed, and in addition to curative treatment options such as surgery or radiotherapy, active surveillance is now considered a suitable treatment option [12–14]. Active surveillance is a treatment option that undertakes regular structured evaluation of prostate cancer disease status. This regular structured monitoring of the disease state is enabled through routine clinical examinations which include prostate-specific antigen (PSA) testing and physical examinations which can include digital rectal examinations (DRE) and imaging and/or biopsies of the prostate [14]. The main aim of active surveillance is to identify progressing or advancing disease that is likely to require curative (also called ‘radical’) treatment whilst the disease remains localised (contained within the prostate gland) [15].

Whilst active surveillance can delay and potentially avoid the quality of life impacts from physical and often ongoing side effects associated with prostate cancer treatments [16], concerns have been raised about the psychological impact of active surveillance as a treatment [17]. Thirty-eight percent of men on active surveillance discontinue in favour of curative treatment, without evidence of disease progression [18], and despite comparable survival outcomes to curative treatment without the associated symptom burden [13]. Men on active surveillance also report ongoing anxiety and uncertainty related to the repeated testing that is required to assess disease status [19], concerns about living with an untreated cancer, including uncertainty about the future and fear for their mortality [15], and the need for greater levels of support to manage their active surveillance treatment [20]. For health care professionals to be able to develop effective interventions to better support men on active surveillance for prostate cancer, a deeper thematic understanding of men’s lived experience is the first step in this process and meta-synthesis can achieve this.

### Objectives

The objective of this literature review and meta-synthesis was to understand the lived experience of men on active surveillance for prostate cancer. Whilst previous studies have explored this, many are largely focussed on Patient Reported Outcome Measures (PROMs) and quantitative surveys [21, 22], initial treatment decision making [23–25], or transition to curative treatment [26] versus the lived experience of being on active surveillance following a diagnosis of prostate cancer. Hence, the focus of this review was on qualitative studies reporting the lived experience of men diagnosed with prostate cancer who had commenced active surveillance and had not yet transitioned to curative treatment.

## Methods

The reporting of this systematic review was guided by the Preferred Reporting Items for Systematic Reviews and Meta-Analyses 2020 statement (PRISMA 2020) [27], and was prospectively registered with the International Prospective Register of Systematic Reviews (Prospero), registration number CRD42023438045.

### Terminology

For the purposes of this review, active surveillance is defined as a treatment that undertakes regular structured evaluation of prostate cancer disease status with the goal of identifying progression whilst curative treatment remains possible [28]. We included studies which used the term ‘watchful waiting’, a process of symptomatic evaluation for cancers not deemed suitable for curative treatments but defined the treatment as aligning with active surveillance goals of treatment.

### Eligibility criteria

Studies were included if they reported qualitative data on (i) the experiences of men diagnosed with prostate cancer who had commenced on active surveillance and (ii) unmet information and supportive care needs of men on active surveillance for prostate cancer. Studies needed to report original research and be published in English.

Studies were excluded if they included participants that had undergone prior treatment for prostate cancer; reported data from patients on watchful waiting (unless results were clearly able to be extrapolated by treatment type); focussed on decision-making regarding the choice to commence or cease active surveillance; reported quantitative data only; were non-empirical publications; and/or were published in a language other than English.

### Search strategy

A systematic search utilising MeSH terms and keywords was conducted on September 1, 2024. CINAHL, Embase, Medline, PsychINFO, and Scopus databases were searched for peer-reviewed literature from the January 1, 2000, to present (Supplementary Table 1). The year 2000 was chosen as active surveillance was first described in the 1990s and patient experiences were first reported in 2002 [29].

### Data collection

All article citations were downloaded from the respective database into Endnote 20 [30]. Duplicate articles were removed using the EndNote ‘Remove Duplicates’ tool and via manual screening. All articles were then transferred to Covidence [31]. Title and abstract screening, and full text review, were conducted by two authors (RB & NH). Differences in inclusion of studies following full text review was discussed between both authors until consensus for inclusion was reached.

### Data extraction

Information from included studies describing author and year, country of study, study type, total number of participants and number of participants on active surveillance, recruitment sampling approaches, age range (and mean) of participants, study aims/focus, and data collection methods were extracted and recorded in an Excel spreadsheet. Themes and findings from the included studies were summarised.

### Study risk of bias assessment

Quality appraisal of all included studies was undertaken independently by two authors (RB, NH) using the Critical Appraisal Skills Programme (CASP) tool [32]. Studies were not excluded based on quality rating.

### Synthesis methods

Raw qualitative data was sequentially and inductively explored for themes using Thomas and Harden’s approach [33]. Categorisation of data was undertaken first using line by line coding of each quote (step 1). This coding was independently undertaken by RB and NH. Coding consensus was reached through discussion. Step 2 involved collecting and connecting these codes inductively to describe linkage. From here, hierarchical branches of codes were established categorising ideas into common and significant perceptions, challenges, and enablers of the lived experience of active surveillance for prostate cancer. In step 3, themes were used to identify the emerging concepts and interpret these in the development of analytical themes retrieved from the collective dataset.

## Results

### Study selection

**Fig. 1:**
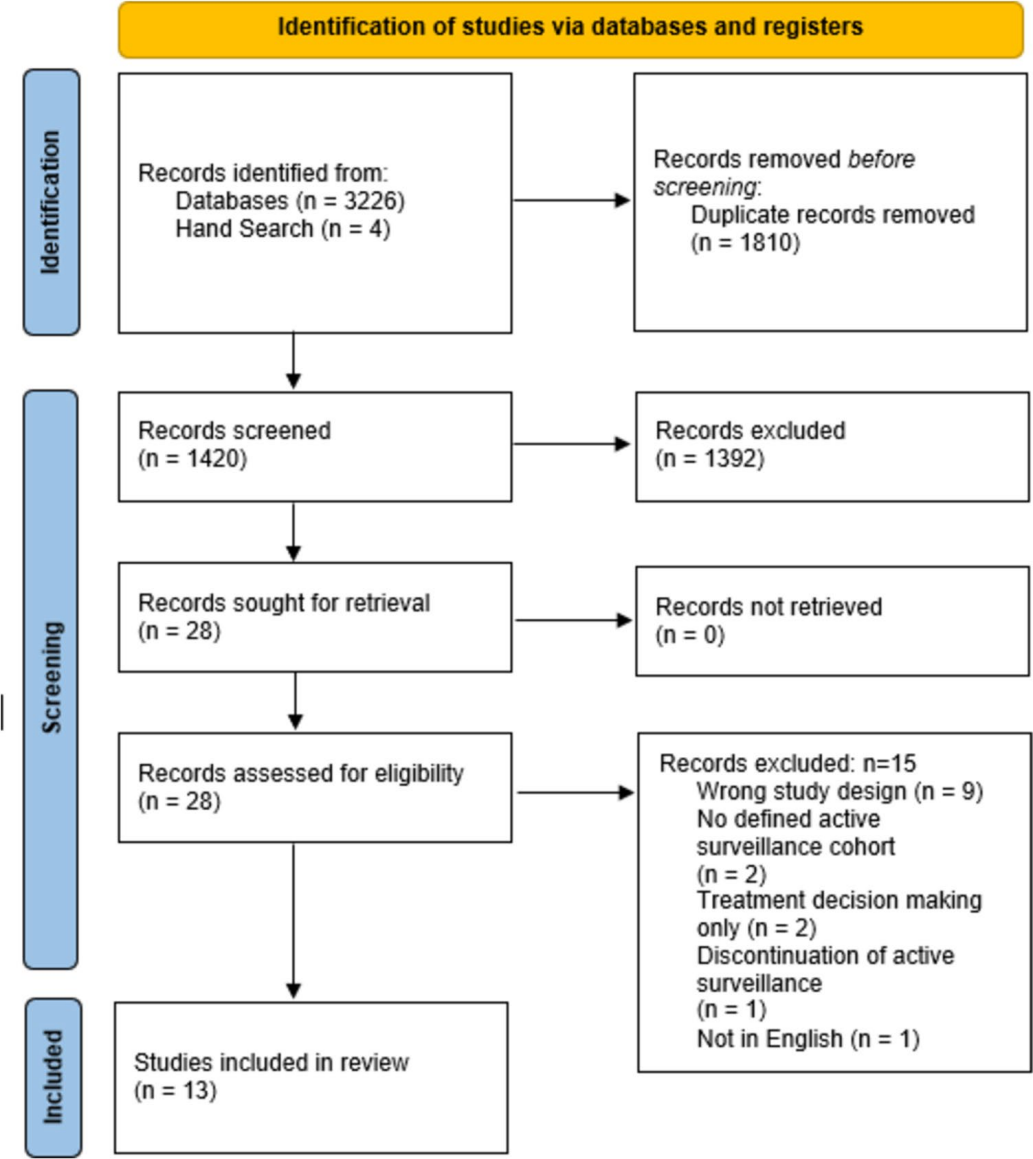
Flowchart of studies through the review process

A total of 3226 articles were initially identified through database searches and hand searching (*n* = 4) (Fig. 1). Following removal of 1810 duplicate articles, 1420 remained for review. Title and abstract reviews were undertaken by two researchers (RB and NH), with 1392 articles not meeting the inclusion criteria. The remaining 28 studies underwent full text review. A further 15 studies did not meet the inclusion criteria, leaving 13 studies [34–46] that explored the lived experience of men on active surveillance for prostate cancer. A summary of included studies is in Table 1.

**Table 1:** Summary of included studies

Author/year/country	Study type/data collection method	Participants	Sampling/settings	Age (years) Mean/(Range)	Time since diagnosis Mean/(Range)	Study aims/focus	Main themes identified
Eymech et al 2021 UK	Qualitative exploratory Semi-structured interviews	Men on active surveillance (*n* = 13)	Purposive through active surveillance clinics in large tertiary hospital	66 (57–74)	4 years (1–7)	Explore the experiences of patients with prostate cancer undergoing active surveillance and describe the effect of active surveillance on their wellbeing	Mental wellbeing - Recognition of impact of diagnosis - Unsettling monitoring cycle - A future problem Social wellbeing Physical wellbeing
Loeb et al 2018 USA	Qualitative Focus groups (*n* = 7)	Men on active surveillance (*n* = 37)	Purposive through electronic records from two clinical sites	Not reported	Not reported	Undertake comprehensive qualitative study of patients and providers to better understand perceptions of existing information sources and recommendations for additional resources, including perspectives about social and other digital media	Themes identified related to informational needs during active surveillance: - More information of Prostate cancer - Greater clarification on difference between active surveillance and watchful waiting - More information on complementary options - Greater variety of resources - More social support and interaction - Verified integrity of information
Mallapareddi et al 2017 USA	Qualitative Focus groups (*N* = 5) Men (*n* = 3) partners (*n* = 2)	*N* = 12 Men on active surveillance (*n* = 12)	Purposive through local cancer registry and academic urologist	61 (47–71)	Median only reported: 1.5 years (0.5–6 years)	Better understand experiences undertaking active surveillance for low risk prostate cancer and why men adopt it	The most common concerns about active surveillance were: - Concerns about reliability of PSA - Pain associated with biopsies - Potential cancer progression Lack of understanding of active surveillance being a recognised management option and not a case of ‘doing nothing’
O'Callaghan et al 2014 Australia	Qualitative exploratory Semi structured interviews (*n* = 35)	*n* = 35 (Patients *n* = 21/partners *n* = 14) *N* = 35 Men still on active surveillance only (*n* = 11) Partners of men still on active surveillance only (*n* = 4)	Purposive through private urology practice, integrative cancer centre and public hospital oncology service	Reported as age ranges only (reported as ≤ 50 to 71 +)	22 months (3–96 months)	Examine men’s and partners treatment decision-making and their experience on active surveillance when it is recommended	- Men and partners both experience and often cope with active surveillance - Active surveillance stressors are endured or inform radical treatment decisions
Kazer et al 2011 USA	Qualitative Focus groups (*n* = 2)	Men on active surveillance (*n* = 7)	Purposive through identification by participating urologist	70 (65–79)	Not reported	Determine the psychological and educational needs of men undergoing active surveillance for prostate cancer	Five categories were identified describing the needs of men (psychological and educational): - Sources of support - Sources of information - Disease monitoring/vigilance - Myths/misinformation/FAQ—accurate information - Health promotion and taking charge
Oliffe et al 2009 Canada	Interpretive description Semi-structured interviews *n* = 25	Men on active surveillance *n* = 25	Purposive Recruited through mail out from two large health centres to eligible patients	68 (48–77)	Less than 2 years (*n* = 22) remaining 3 Not reported	Describe the range of behaviours used by men on active surveillance as an interim step to suggesting specific psychosocial interventions with which men are likely to engage	Two distinct strategies were identified that describe men’s self-management to overcome active surveillance related uncertainty: - Living a normal life (protective mechanism—downplay uncertainty) - Doing something extra (through research and adjunct therapies)
Davison et al./2009 Canada	Qualitative (phenomenological approach) Semi-structured interviews *n* = 25	Men on active surveillance *n* = 25	Purposive through identification of eligible patients by doctors at 2 large tertiary hospitals	66 (48–77)	< 1 year *n* = 13 1–2 years *n* = 9 > 2 years *n* = 3	Identify and describe how men arrived at their decision to go on active surveillance as a preliminary step to identifying what types of resources and supports might be of future benefit to them	- Clinician description of prostate cancer influenced perceptions of seriousness - Men trusted physician to recommend best treatment - Men chose active surveillance because physician considered this the best approach - Accepting advice from friends depended on whether friends had prostate cancer experience - Fear of spread was real despite regular follow-ups and reassurances - Men may try to carry on as usual and to minimise cancer status
Mader et al 2017 USA	Qualitative Semi-structured interviews *n* = 15	Men on active surveillance *n* = 15	Purposive from two academic institutions	Not reported	Not reported	Investigate the primary coping mechanisms for men on active surveillance with a specific focus on how men interact with their social network, and they negotiate stress and uncertainty of diagnosis and treatment approach	- Selection of active surveillance was part of coping as it allows men to live a normal life - Social support was relied upon to assist with coping - Trust in the medical teams assisted coping
Berger et al 2014 USA	Qualitative (mixed methods) Semi structured interviews	Men on active surveillance *n* = 14	Purposive from active surveillance program at tertiary hospital	Not reported	Not reported	To better understand men’s experiences on active surveillance	The experiences of active surveillance follow-up schedule were seen as: - Stressful and anxiety causing - Relieving and confidence building in treatment success Men leaving active surveillance without signs of disease progression described reasons for this - Concerned about the longevity of active surveillance - Burdened by ongoing biopsies and follow-up
Bailey et al 2005 USA	Qualitative Semi structured interviews *n* = 10	Men on active surveillance *n* = 10	Purposive form urology clinic in larger tertiary hospital	64–88 (mean Not reported)	4–12 months	To explore the problems and uncertainties of older men with prostate cancer and the strategies they use to manage these problems after undergoing watchful waiting* *Watchful waiting terminology used in study; however, description of treatment aligns with active surveillance	- Uncertainty about disease and treatment—defining feature of uncertainty was that prostate cancer offered few signals (symptoms) as patients tried to monitor disease - Danger appraisal—there are many treatment alternatives, and a lack of clear guidelines on appropriateness of treatment make appraisal of danger in treatment decision making unique to prostate cancer patients - Opportunity appraisal—viewing decision as opportunity to successfully manage anxiety through work, self-care, keeping options open, use of alternative medications and prayer
Mroz et al 2013 Canada	Qualitative Semi-structured interviews *n* = 25	Men on active surveillance *n* = 25	Purposive Recruited through mail out from two large health centres to eligible patients	68 (< 65–70 >)	< 1 year–2 years >	Describing the connection between masculinities and patient's perspectives of male patient-physician communication in the context of active surveillance	Therapeutic communications - Patient-physician communication featured prominently—participants reported accepting the clinical communication style and recommendations - Understanding the specifics of active surveillance was seen as the key to sustaining on treatment - Unhelpful communication was seen as crucial for lacking confidence in doctor Threat-based communications: - The ‘threat’ experience of poor communication from physicians resulted in lack of understanding, increased rumination about the risk of cancer spreading and dissatisfaction about the lack of details regarding cancer as well as monitoring protocols
Volk et al 2014 USA	Qualitative Semi structured interviews *n* = 15	Men on active surveillance *n* = 15	Purposive from prostate cancer clinic in tertiary hospital	62.6 (49–72)	6–18 months	To understand the views of active surveillance patients diagnosed with localised prostate cancer	- Active surveillance is an organised, supportive process - Active surveillance prolongs current good health - The cancer is low risk and active surveillance allows time to decide about treatment - Active surveillance allows the avoidance of side effects of treatment - Physician recommendation about active surveillance is important - Men can feel a need to justify the decision to others
Donachie et al 2020 Netherlands	Qualitative Semi-structured interviews *n* = 17	Men on active surveillance *n* = 17	Purposive from two participating hospitals	67 (54–76)	< 1–120 months	To identify the psychosocial support needs of prostate cancer patients during active surveillance	- The diagnosis of cancer results in impacts of fear and confrontation about own mortality - Confidence in the expertise and experience of their physician and an emotional connection play an important role - Effective coping strategies reduced the psychosocial burden associated with active surveillance - Repeat prostate biopsies—cause anxiety and uncertainty - Men have a latent fear of disease progression and are especially concerned about the presence of undetected metastases - There is a strong need for information (reliable, disease specific) in the first 2 weeks up to 4 months post diagnosis - There is insufficient or inadequate information regarding scientific developments and innovations, available treatment options, recommended lifestyle modifications - Recurring anxiety and uncertainty occurred in the majority of participants

*Active surveillance* active surveillance, *NR* not reported

### Study characteristics

#### Study settings and designs

Seven of the 13 studies were conducted in the USA [35–37, 41–43, 45], three studies were set in Canada [39, 40, 44], with one each from the UK [34], Australia [38], and the Netherlands [46]. All studies were conducted between 2005 and 2021. In eight of the studies participants were recruited through clinics, patient databases, or medical records from large tertiary treatment centres [34, 37, 39, 40, 42, 44–46]. Two studies recruited participants through participating urologists’ patient records [36, 43]. One study used the urologists practice database of suitable patients in addition to a local cancer registry [35]. In one study, it was not clear where the patients were recruited, although it identified that interviews were conducted with patients through two academic institutions [41]. In the remaining study, recruitment was through a private urology practice, an integrative cancer centre or a public hospital active surveillance clinic [38].

All studies were qualitative in design with the exception of one that employed a mixed methods approach [42].

#### Participants

Recruitment of participants was purposive in all studies. The numbers of participants ranged from 7 to 37. Age of participants ranged from 47 to 88, with mean age ranging from 61 to 70. Four of the studies did not report age range or mean age [37, 38, 41, 42].

#### Data collection methods

Semi-structured interviews were used for data collection in ten studies [34, 38–46]. The remaining three studies utilised focus groups for their data collection [35–37].

### Risk of bias in studies

The quality of all included studies per the CASP tool [32] was high (refer Supplementary Table 2). The aim of the research was clearly identified in all studies and appropriate methodology was utilised to undertake qualitative research. Ethical considerations were evident in all studies and recruitment and data collection approaches were well articulated. Findings from each study were clear and the value of the research was evident. The ability to understand the relationship between the researcher and participants was only evident in three of the studies [34, 36, 38].

### Themes

**Fig. 2:**
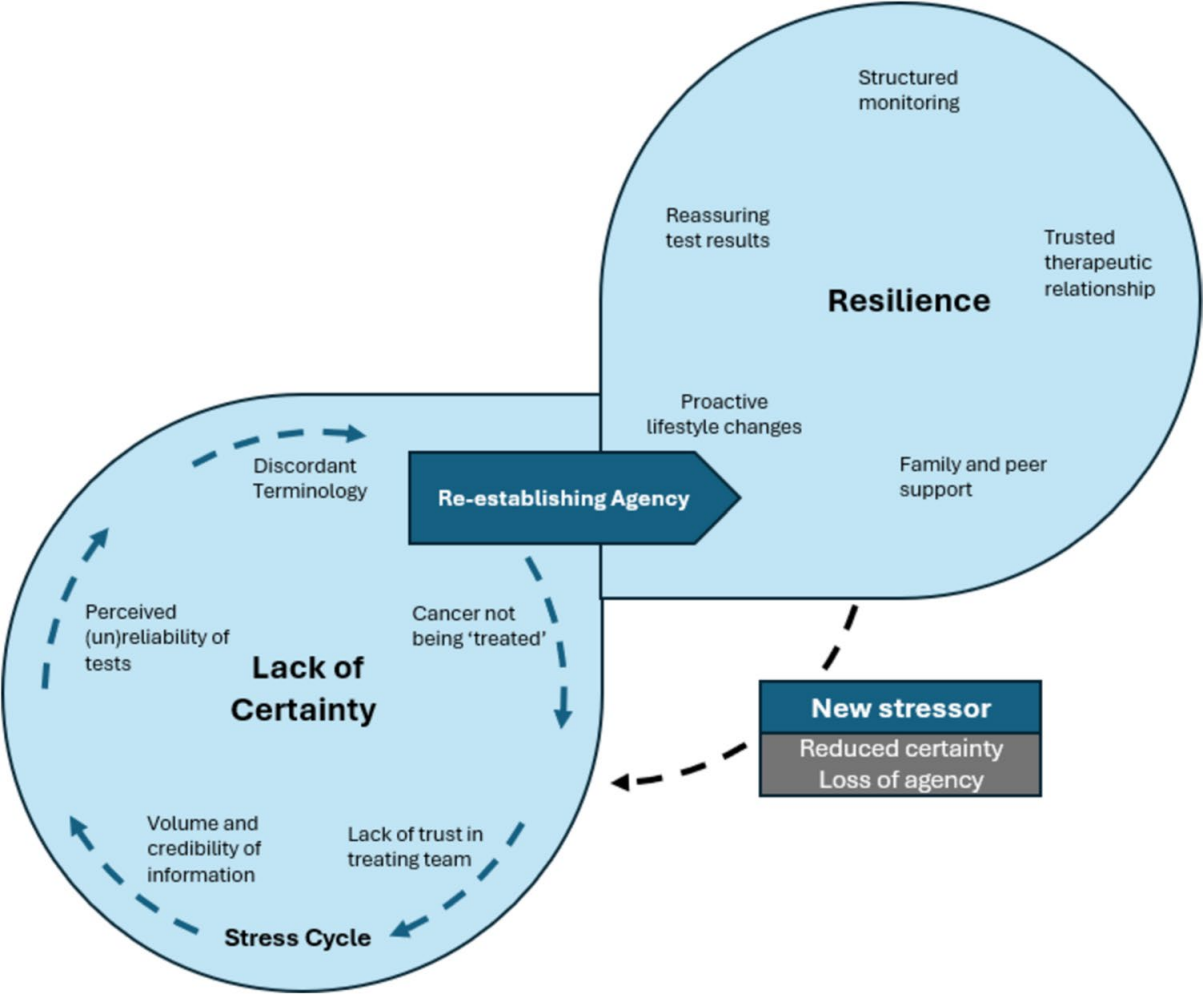
Conceptual diagram of the lived experience of active surveillance for men with prostate cancer

Two overarching analytical themes were identified in this meta-synthesis: (i) men on active surveillance live with a lack of certainty; and (ii) re-establishing agency (i.e. a feeling of control over actions and their consequences [47]) drives resilience and facilitates confidence in active surveillance (Supplementary Table 3). Lack of certainty on active surveillance is derived from men feeling a loss of control over their health and/or lives as they continue to live with an essentially untreated cancer. This lack of certainty induces a cyclical stress response of ongoing worry and anxiety and a resultant loss of agency, which further drives the stress cycle. However, re-establishing agency alleviates the stress response, promotes resilience, and facilitates confidence in active surveillance as a treatment choice (Fig. 2).

#### i. Men on active surveillance live with a lack of certainty

##### My cancer is not being treated; it’s still growing inside me. It worries me

Men live with a lack of certainty whilst on active surveillance driven by ongoing awareness of an ‘untreated’ cancer remaining in their body [46] describing that this worry never ‘leaves their mind’ [36, 46]. Clinicians’ explanations of low-grade, low-aggressive, or slow-moving disease do not ameliorate these concerns [40, 44], and any time cancer is mentioned men report feeling frightened [43].

##### The tests aren’t reliable, what if it gets away?

Additionally, men are worried that their cancer will progress undetected, and their opportunity for cure will be missed [35]. The fear of undetected disease progression is perpetuated by men linking any new physical symptoms directly to their cancer and seeing symptoms as signs of advancing disease [43], aided by a lack of clear information as to what indicates if progression is occurring [37]. The monitoring tests associated with active surveillance do not drive optimism in treatment. Men are dubious as to their efficacy and reluctant to be reassured by results as they lack trust in the tests to adequately detect disease progression [38, 42, 46]. The routine follow-up required as part of active surveillance exacerbates these worries with men detailing the anxiety they suffer at the time of follow-up [34, 39] and waiting for the results [34]. Collectively, these factors lead men to question their choice of an observational strategy versus curative treatment [43].

##### I don’t have any trust in my treating team

Adding to their lack of certainty, the trust men develop in their treating team influences their experience of active surveillance. Men describe a feeling of disconnect, no rapport or connection [44], and a lack of responsiveness to questions [44] as being detrimental to developing trust in their treating team, and establishing confidence in active surveillance to safely monitor their disease. Men have also detailed that conflicting information and advice from health care team members impedes trust in the team [38].

##### There is so much information out there, how do I know what I can trust?

Men actively seek information on their disease and their treatment, beginning early following diagnosis [46]. Men report being presented with an abundance of information on prostate cancer [36]; however, information is not always easy to understand [37] and that the information available can often be contradictory and confusing [35]. Men are concerned about the ability to verify the integrity of information available and seek sources that can be trusted and reliable [37], provide clear information relevant and specific to active surveillance, and detail new developments related to active surveillance [36].

##### Discordant terminology

- **Do I have cancer or not?**
  Discordant terminology used by treating teams at diagnosis and when describing active surveillance contributes to men’s lack of certainty. Men describe members of the treating team avoiding the term ‘cancer’ altogether at diagnosis, instead describing a presence of ‘…some atypical cells’ [44] or indications of cancer [40] and telling men ‘…you don’t have a problem’ [38].
- **Cancer should be treated.**
  Upon commencing active surveillance, men explain that treating teams have described active surveillance as ‘…not doing anything’ [44] or ‘…leaving it as it is’ [44] rather than a structured treatment regime designed to monitor disease progression, with the end goal to offer further curative treatments [48]. This can result in men not believing that they are on a recognised treatment strategy [40]. Men also detail pressure from family and friends to properly ‘treat their disease’ adds to their stress as they feel required to defend their decision to commence and continue active surveillance [36].
- **What am I waiting for? **
  Furthermore, men describe terminology being used by treating teams that is discordant with the clinical description of active surveillance, terms such as ‘watch and wait’, ‘wait and see’, and ‘watchful waiting’ [35–37, 44], which can result in men not comprehensively understanding their treatment and further adding to confusion and lack of certainty.

#### ii. Re-establishing agency drives resilience and facilitates confidence in active surveillance

Men seek to maintain agency in their treatment, i.e. having the capacity to initiate and manage actions in response to awareness and ownership of health-related needs [8], enabling them to feel some control. This in turn promotes resilience, the successful adaptation to challenging life experiences through behavioural flexibility [49], to cope with the lack of certainty they experience with active surveillance and promotes confidence.

##### They are going to watch me closely, on a structured program

The knowledge that they are on a surveillance program with structured specifics of when and what follow-ups are required [34, 39, 41, 45], and that the treating team have the skills to identify and treat progressing disease [45], promotes confidence for men and supports their ability to develop resilience to the lack of certainty they face, and adhere to their chosen treatment [40].

##### Trust in the treating team and test results promotes confidence

In contrast to the impact of sub-optimal therapeutic relationships, feelings of trust in their treating team enhances men’s resilience, supporting them to feel more confident in active surveillance [35]. Factors that promote trust in the treating team for men include professional qualifications and clinical experience [38, 44], feeling that their team are good at what they are doing and [41] know them and their medical history [35], reviews, and recommendations from peers and/or websites [37], and positive experiences from team members manner and style [44] all leading to men having trust in their team [35, 36, 41]. Consistency in advice and recommendations from clinicians and treating teams is also reassuring for men, adding to their confidence in active surveillance [35, 41]. Feeling they have received information that meets their needs also promotes agency for men, supporting their resilience and confirming they have chosen the correct treatment. Men describe that information provision to the level of their need and understanding is reassuring, and that their treating team is a great source of information to them [37, 46]. Men’s confidence in their active surveillance is further buoyed by favourable clinical results as they provide men with objective measures that their treatment is successfully monitoring their disease [35, 41].

##### Family and peer support

Family and peer support also promotes men’s resilience in managing lack of certainty. Men detail support from family and being able to talk to other men as being beneficial in this process, affirming that active surveillance is the correct treatment for them [36–38].

##### What can I do to help myself?

To further promote agency and support resilience men proactively seek out information to improve understanding of their diagnosis and chosen treatment. Information seeking commences early [46], and can be ongoing, to understand changes as they occur [37]. Some men describe a need to ruminate over the information they find to ensure they understand their treatment and risk [46] and are comfortable with their decisions. Information seeking is not solely undertaken by men, with some men describing family members pursuing information and passing on findings to them [36].

In addition to information seeking, men also detail proactive lifestyle changes they make to benefit not only their general health, but also in the hope that it may impact cancer progression [46]. Men seek tips and advice on diet and lifestyle modifications that they can make [46], supported by the understanding that there is ongoing messaging that eating better and exercising more is advisable. Men are keen to ascertain if there is any advice, ‘…proven or not…’ [37, 39] on the correlation this can have on cancer progression [37] with some men crediting their diet with improving test results [46]. These lifestyle changes are not all driven by men; wives and partners are credited by many men as being the driver [41] in making lifestyle changes and being their biggest supporters in coping on active surveillance and making proactive health changes [41].

## Discussion

This meta-synthesis has identified that men’s experience of active surveillance is dominated by a lack of certainty. This lack of certainty is derived from men feeling a lack of control over their situation resulting in men enduring stress [50]. In the face of this stress, men look to regain agency in their treatment to enable them to feel some control over their health. Agency is boosted by an understanding of how their cancer will be monitored, reassuring test results, proactivity (active information seeking, lifestyle, and dietary changes), trust in their treating team, and family and peer support. These actions, in response to the lack of certainty men experience on active surveillance, can alleviate the associated stress and lead to better psychological outcomes [51]. If new stressors emerge (e.g. change in test results, lack of trust in treating team), men on active surveillance face the potential of returning to a lack of certainty and loss of agency, resulting in a return to the stress response.

Notably, masculinity is influential in men’s responses to illness and may impact their help-seeking behaviours [52]. Masculine domains of note, when assessed in the context of chronic disease, include (i) strength, (ii) sexual importance/priority, (iii) family responsibilities, (iv) emotional self-reliance, (v) optimistic capacity, and (vi) action approach [52]. The stress cycle response adversely impacts the masculinity domain of optimistic capacity and unconsciously can result in men having the same emotions they experience with anger and fear [53]. In contrast, strength-based masculinities can be of benefit by men adopting problem solving and making proactive changes [54]. Hence, action-oriented coping strategies as identified in this meta-synthesis, such as information seeking and proactive lifestyle changes, are consistent with masculine values and emotional self-reliance and connect to the importance of maintaining personal agency [52].

Men’s trust in their treating team is an essential driver in establishing confidence in treatment and regaining agency. Understanding the active surveillance protocol itself and how the various tests being utilised identify progressing disease is key to establishing confidence. However, there is currently little clinical consensus for either of these factors. A review of contemporary worldwide practices in active surveillance for prostate cancer identified significant variations between protocols including the criterion used to identify patient suitability, and a lack of consensus regarding frequency for PSA testing, DRE, re-biopsy, and general health assessments [18, 55]. A clear understanding of what indicates progression of disease is important to men; however, indicators used to identify this also vary between settings [18]. Identification of clinical progression markers lack universal agreement [18], and, whilst there are numerous urological guidelines for active surveillance management globally, variations in clinical practice persist [13, 14, 18, 55]. Discussions with men at commencement of treatment and at subsequent reviews relating to the status of their disease, and the identifiable criterion that would indicate progression, may assist men in regaining agency.

Problematically, confidence in active surveillance may also be undermined early in the treatment choice based on the utility of PSA testing pre and post diagnosis. Pre-diagnosis, the ability of PSA as a biochemical marker to identify or diagnose cancer comes into question for men as PSA is prostate specific, not cancer specific [56]. Due to this specificity, PSA elevation alone does not provide enough evidence to inform a cancer diagnosis; instead, it is an indicator of cancer potential amongst a multitude of other possible causative factors [57]. However, following a diagnosis, PSA is utilised globally as a key criterion as an indication of cancer progression [18]. Men, in the process of their diagnosis, will likely have been advised at the commencement of investigations that an elevated PSA is not definitive enough to provide diagnosis as it is not cancer specific, however post diagnosis are advised to rely on this same measurement to identify disease progression.

Men report clear gaps in the availability and quality of accessible and easily understandable information specific to active surveillance, particularly at the start of treatment, undermining their confidence in active surveillance and adding to their lack of certainty. In addition, clinicians’ use of discordant terminology related to both newly diagnosed prostate cancer and active surveillance itself compounds men’s lack of certainty. A key finding of this meta-synthesis is the often interchangeable and/or incorrect use of ‘watchful waiting’ instead of active surveillance. In contrast to active surveillance, watchful waiting is a process of symptom evaluation which often incorporates monitoring of PSA but does not routinely include regular/routine imaging and biopsies to assess disease progression [13]. The key difference between these approaches is that men on active surveillance retain the option of curative treatment in the event of disease progression, whereas men on watchful waiting have been deemed not suitable for curative treatment of their disease. The focus of watchful waiting is symptom management, either locally or systemically as a result of progression of disease and treating these symptoms to maintain quality of life [13]. Moreover, there is coupling of active surveillance and watchful waiting cohorts when reporting key research findings, including incidence and management of prostate cancer, symptom bother and treatment side effects [17, 58], thus limiting the usability of population data for both cohorts.

Terms used to describe active surveillance, including ‘wait and see’ and ‘watch and wait’, are also discordant with the clinical definition and goal of active surveillance. Active surveillance is a recognised, and recommended treatment [18]; however, these descriptions contribute to a lack of certainty. Health care providers are cognisant of the differences in these treatments; however, patients are largely not. The use of interchangeable and discordant terminology creates the potential to inhibit men’s understanding and therefore limit their commitment to the required vigilance on such a treatment. In addition to incorrect language relating to active surveillance, risk minimisation and avoidance of cancer as a word when discussing a diagnosis was also evident, further impacting men’s ability to feel fully informed of their diagnosis and risk classification. Health communication and literacy are recognised as important constructs in health care [59], previous studies investigating communication in cancer have identified that many participants did not fully understand the language used by surgeons and cancer specialists [60]. Whilst these findings are not unique to prostate cancer or men on active surveillance, they do highlight the importance of clear and consistent terminology when caring for this cohort of men.

The therapeutic relationship the man has with his treating team is integral in establishing and maintaining confidence as they commence and stay on active surveillance. Men’s trust in their treating team is underpinned by a feeling that their team are experts in their field and know them well in the context of their care. Patients’ trust is enhanced by the clinicians perceived competence, honesty, and patient-centred behaviour [61]. For men with prostate cancer specifically, perceived closeness with and trust in the physician are associated with patients feeling they have received better treatment, particularly when active surveillance is recommended [62]. In practice, this would support exploring patient concerns and information needs, finding common ground on diagnosis and continuing to enhance the relationship [63]. Conversely, a lack of responsiveness, failure to answer questions, or feeling of a lack of connection was identified as opposing the establishment of a therapeutic relationship and decreased the value men placed on information provided to them. The absence of a therapeutic relationship is associated with higher information-seeking behaviour [64]. High uncertainty increases worry and the risk that men will discontinue with active surveillance, as evidenced by the 38% of men discontinuing already without any clinical indication [18]. Provision of support in responding to and managing this uncertainty is essential [65].

In this meta-synthesis, we identified that men’s ability to develop trust in their treating team benefits their experience with active surveillance treatment and also influences men’s information seeking. Patients generally desire basic information following a diagnosis of cancer; however, not all want more detailed information [66]. Assessing and addressing information needs is positively associated with cancer related health outcomes [67]. However, meaningful information specific to active surveillance is not easily accessible, and information that was found was not easily understood. The information-seeking behaviour of patients has increased over time [68]; however, despite this, health care providers remain a key source of information for patients [68].

### Implications

Studies included in this systematic review and meta-synthesis report on patients that have been on active surveillance for a wide variation of time frames. It is well established that there is increased distress for men diagnosed with prostate cancer, particularly in the first 12 months regardless of treatment type [7, 8]. Whilst this meta-synthesis has provided insights into the lived experiences of men on active surveillance for a varied timeframe, the experiences of men in the first 12 months where prostate cancer–related distress is known to be higher are not clear. However, what is evident is that addressing the following key considerations will best support men on active surveillance: (i) awareness of masculinity and its impact on help-seeking behaviours; (ii) understanding the importance of the therapeutic relationship and trust in the treating team for men; (iii) provision of tailored evidence-based information to support men’s understanding of their diagnosis and active surveillance treatment protocol; and (iv) use of consistent terminology that aligns with the clinical definition of active surveillance. Further research to better understand men’s needs during this timeframe and the specific type of support they feel they need across the active surveillance trajectory is warranted.

### Limitations

Whilst this study has provided new insights into the lived experience of men on active surveillance, there are some limitations. Studies were limited to those published in English. Some studies may have been missed as they used terminology that does not align with the clinical definition of active surveillance and therefore not been identified as part of the search strategy.

## Conclusion

This meta-synthesis has identified that the central stressor or challenge for a man with prostate cancer on active surveillance is lack of certainty. Men respond to this stressor in different ways and the relationship with the healthcare team can help or hinder men’s experiences. The development of confidence is underpinned by the ability to regain agency. Active coping and problem-solving appear to be key drivers of supporting agency and promoting resilience.

Our goal as health professionals is to recognise and address unmet needs and reduce the risk of men prematurely discontinuing active surveillance without clinical indication. It is therefore a research priority to understand the needs of these men in the context of masculine values and help seeking to inform interventions that assess and address unmet needs and enable tailored, person-centred care.

## Supplementary Information

Below is the link to the electronic supplementary material.

Supplementary Table 1: Sample search strategy (DOCX 18.7 KB)

Supplementary Table 2: Quality Appraisal (DOCX 21.1 KB)

Supplementary Table 3: Analytical themes and sub-themes (DOCX 29.2 KB)

## Data Availability

No datasets were generated or analysed during the current study.
