# Supplementary material for: The lived experience of active surveillance for prostate cancer: a systematic review and meta-synthesis
Source: J Cancer Surviv. 2025 Feb 12;20(4):1462–79. doi: 10.1007/s11764-025-01748-x (PMC13375763; doi:10.1007/s11764-025-01748-x)
Supplement: Supplementary file 1 — Supplementary Table 1: Sample search strategy (DOCX 18.7 KB) [file 11764_2025_1748_MOESM1_ESM.docx]

**Supplementary information**

**Supplementary Table 1: Sample search strategy**

Database: Embase <2000 to 2024 July 20>
Search Strategy:
--------------------------------------------------------------------------------
1 prostate cancer.mp. or exp prostate cancer/ (304826)
2 active surveillance.mp. or exp active surveillance/ (17811)
3 watchful waiting.mp. or exp watchful waiting/ (8255)
4 patient education.mp. or patient education/ (135612)
5 supportive care.mp. (40392)
6 information*.mp. (2423828)
7 psychosocial*.mp. or exp psychosocial care/ (188870)
8 coping.mp. or exp coping behavior/ (127567)
9 unmet needs.mp. or health care need/ (49512)
10 survivorship.mp. or survivorship/ (27789)
11 2 or 3 (24842)
12 4 or 5 or 6 or 7 or 8 or 9 or 10 (2873950)
13 1 and 11 and 12 (1162)

Search for: 1 and 11 and 12

Results: 1162
