# Supplementary material for: The lived experience of active surveillance for prostate cancer: a systematic review and meta-synthesis
Source: J Cancer Surviv. 2025 Feb 12;20(4):1462–79. doi: 10.1007/s11764-025-01748-x (PMC13375763; doi:10.1007/s11764-025-01748-x)
Supplement: Supplementary file 2 — Supplementary Table 2: Quality Appraisal (DOCX 21.1 KB) [file 11764_2025_1748_MOESM2_ESM.docx]

**Supplementary information**

**Supplementary Table 2. Quality Appraisal**

| **CASP Domains^[[1]](#footnote-1)^** | **Eymech et al / 2021** | **Mallapareddi et al / 2017** | **Kazer et al / 2011** | **Loeb et al /  2018** | **O'Callaghan et al / 2014** | **Oliffe et al / 2009** | **Davison et al / 2009** | **Mader et al / 2017** | **Berger et al / 2014** | **Bailey et al / 2005** | **Mroz et al / 2013** | **Volk et al / 2014** | **Donachie et al / 2020** |
| --- | --- | --- | --- | --- | --- | --- | --- | --- | --- | --- | --- | --- | --- |
| Was there a clear statement of the aims of the research? | Y | Y | Y | Y | Y | Y | Y | Y | Y | Y | Y | Y | Y |
| Is a Qualitative methodology appropriate? | Y | Y | Y | Y | Y | Y | Y | Y | Y | Y | Y | Y | Y |
| Was the research design appropriate to address the aims of the research? | Y | Y | Y | Y | Y | Y | Y | Y | Y | Y | Y | Y | Y |
| Was the recruitment strategy appropriate to the aims of the research? | Y | Y | Y | Y | Y | Y | Y | Y | Y | Y | Y | Y | Y |
| Was the data collected in a way that addressed the research issue? | Y | Y | Y | Y | Y | Y | Y | Y | Y | Y | Y | Y | Y |
| Has the relationship between the researcher and participants been adequately considered? | Y | Can't Tell | Y | Can't Tell | Y | Can’t Tell | Can’t Tell | Can’t Tell | Can’t Tell | Can’t Tell | Can’t Tell | Can’t Tell | Can’t Tell |
| Have ethical issues been taken into consideration? | Y | Y | Y | Y | Y | Y | Y | Y | Y | Y | Y | Y | Y |
| Was the data analysis sufficiently rigorous? | Y | Y | Y | Y | Y | Y | Y | Y | Y | Y | Y | Y | Y |
| Is there a clear statement of findings? | Y | Y | Y | Y | Y | Y | Y | Y | Y | Y | Y | Y | Y |
| How valuable is the research? | Valuable | Valuable | Valuable | Valuable | Valuable | Valuable | Valuable | Valuable | Valuable | Valuable | Valuable | Valuable | Valuable |

1. 1. Critical Appraisal Skills Programme (2022). CASP Qualitative Checklist [online] Available at: <https://casp-uk.net/casp-tools-checklists/>. [↑](#footnote-ref-1)
