# Supplementary material for: The lived experience of active surveillance for prostate cancer: a systematic review and meta-synthesis
Source: J Cancer Surviv. 2025 Feb 12;20(4):1462–79. doi: 10.1007/s11764-025-01748-x (PMC13375763; doi:10.1007/s11764-025-01748-x)
Supplement: Supplementary file 3 — Supplementary Table 3: Analytical themes and sub-themes (DOCX 29.2 KB) [file 11764_2025_1748_MOESM3_ESM.docx]

**Supplementary information**

**Supplementary Table 3: Analytical themes and sub-themes**

| Analytical themes | Sub-themes | Illustrative quotes |
| --- | --- | --- |
| Lack of Certainty | My Cancer is not being treated; it’s still growing inside. | “Everyone can tell me.. you will live to be 95 with this disease [..] That cancer is still in my body.” (Donachie et al., 2020)  It is never away, it never leaves my mind. You know it is there..” (Donachie et al., 2020)  It was always in the back of my mind (Kazer et al., 2011)  …I mean it sticks with you, cancer is cancer, I don’t care whether its low grade or not and nobody could tell me that there’s no growth there, there’s no, um, spreading of the cancer, they can’t tell me that. (Mroz et al., 2013)  Well, that [dying from a heart attack] really don’t bother me as bad as thinking about cancer.… Anytime that cancer is mentioned, it’s frightening. A heart attack or stroke or something doesn’t worry me as much as knowing that I’ve got this. (Bailey et al., 2004)  slow-moving turtle [40] |
|  | The tests aren’t reliable, what if it gets away? | My “fear” is that it (cancer) would get away. You’d miss your window of opportunity’. (Mallapareddi et al., 2017)  I would like to know what progresses the cancer, because right now I’m pretty ignorant about the whole thing. (Loeb et al., 2018)  Since this spring my PSA went up 1 point. [..] that makes me think. It concerns me.” (Donachie et al., 2020)  …just because those biopsies are not showing the cancer cells and so forth, in all probability it's in there. It's just that the biopsies are not picking the heat up … it was in my mind, maybe not constantly, but it was there often enough—to the point where, you know what, enough is enough. (Berger et al., 2014)  Every time (each appointment) it is like coming back from the dead because when the results are in "I feel like I'm basically back to normal and life is fine. (Oliffe et al., 2009)  It’s a little scary…...every time I come up to my six month visit…I have a little bit of anxiety and then we do the digital and everything is fine and the PSA is fine......phew...got another six months. So, I sort of live six months at a time basically. (Oliffe et al., 2009)  I don’t worry about it on a day-to-day basis or month-to-month basis. Every six months when I go to my check-up appointment, I get worried about it… (Eymech et al., 2022)  The worst bit was the actual waiting for the results. This plays tricks on your mind. (Eymech et al., 2022)  PSA is useless … biopsies are just not good for you.” … Why would one test come back and give me a very high Gleason count and then the next biopsy actually says there’s nothing there? … I’ve asked these questions but nobody seems to answer them. They … say, “No, no, you are fine.” … I keep saying to my wife, “Should I go and get another opinion … the best of three?” (O'Callaghan et al., 2014)  I noticed that every time I went to the bathroom, I was wondering if it [prostate cancer] was any worse. There were times when urinating wasn’t comfortable or the stream was very slow getting started. I was constantly concerned…was it getting worse. (Bailey et al., 2004)  Well, it’s always in the back of your head. Any time I have like a groin pain, sure, you always wonder if it’s maybe something related to the prostate cancer. (Bailey et al., 2004)  I fell and hurt my hipbone… [I got] sore, and it bothered me, and I said, reckon [is] that [pain] coming from that [prostate cancer]. I don’t know where it’s coming from. (Bailey et al., 2004)  ... I just don’t know if I’m doing the right thing or not, just based on my age – I’ll be 70 years old in May. But I just don’t know to be honest with you. It’s really a major decision. Of course, I still don’t know if I’m doing the right thing, by observing. (Bailey et al., 2004) |
|  | I don’t have faith in the team. | I didn’t have a good rapport with that doctor and I don’t put a lot of stock in what that doctor said, I didn’t feel like we were connecting in any kind of a meaningful way. (Mroz et al., 2013)  I asked him (doctor) if there was any result back from the biopsy and he said he would find out, well I never heard back. (Mroz et al., 2013) |
|  | There is so much information out there, how do I know what I can trust? | There’s all kinds of sources you can get here at the hospital, booklets and pamphlets and there’s different magazines you read and you watch the news… and you get information there, so, I mean, any place you hear about Prostate Cancer, you hear other people talking about it, you know… (Kazer et al., 2011)  There’s a lot of jargon, and it’s putting all the pieces together. Remembering what the Gleason score is. All I remember is that it exists and higher is bad. (Loeb et al., 2018)  …one study read says that PSA’s valid. The other says, eh, it’s not so good. (Mallapareddi et al., 2017)  You need reliable sites. There’s Web MD, there’s …I can’t remember the names. So you can go to reliable sites and they have certain structures with the way they give you the information. (Loeb et al., 2018)  It seems like there should be one or two specific websites like the Hopkins website that I also watch that you can go to because there’s so much information and they can narrow it to 2 or 3 sites or like maybe the site that you’re developing [is like]. The issues that I have with sites is that it is conflicting and you hear from one site and one Doctor and they would recommend something, then, a month or two later, you hear another recommendation and that’s where the confusion comes in and that’s where the frustration comes in and you say, ‘‘O.K. let’s get rid of it. (Kazer et al., 2011) |
|  | Discordant Terminology |  |
|  | Do I have cancer or not? | He (doctor) didn’t even use the word cancer . . . One sample that has an, or some atypical cells but he didn’t say they were . . . he stayed away from the word cancer and they don’t show as being cancerous... (Mroz et al., 2013)  Indications of cancer in the prostate and that you have a free ticket right now, it’s a low-risk cancer, and let’s wait and see. (Davison et al., 2009)  …one urologist could say, 'you’ve still got cancer', whereas another says, 'You don’t have a problem'…The most difficult thing (he added) was getting reliable and personally relevant information. (O'Callaghan et al., 2014) |
|  | Cancer should be treated. | It wasn’t, uh, a big long conversation. They (doctors) say it’s not that bad, yeah, so they’re not going to do anything on that, we’ll leave it as it is. (Mroz et al., 2013)  “If it is cancer, why can’t it be treated? (Davison et al., 2009)  My Brother was concerned that I was not doing anything about the Prostate Cancer and so, he tried to enlist my Sister to get me to do something (Kazer et al., 2011) |
|  | What am I watching and waiting for? | So, basically when I first had this, by it being so small, and that treatment and stuff like that was so invasive. You know he’s, like, quality of life will change. So he just told me to, we’ll do a wait-and-see, and so I did that approach. (Mallapareddi et al., 2017)  So I said I’ll just wait and see. I took the wait and see process because if it get any worse, I’m gonna know what to do when the time comes. (Mallapareddi et al., 2017)  I mean, my “biggest influence” of watch and wait was the urologist’. (Mallapareddi et al., 2017)  ...she (his physician) initially encouraged me to wait and watch. That was kind of where she, she said, you can wait and watch. (Mallapareddi et al., 2017)  I don’t think there was any real discussion about it, it was just ‘here, this is the best thing for you at this point is to wait and see what happens. (Mroz et al., 2013)  I heard about it [AS] just a couple of months ago… until then I didn’t know there was a difference, I didn’t even hear about AS it was just WW. (Loeb et al., 2018).  I have a problem finding the Watchful Waiting areas in the websites. It seems like there’s more and more that are getting into the Watchful Waiting mode. So, I’m encouraged by that because I’m constantly searching. (Kazer et al., 2011).  My doctor explained to me about the “watchful waiting,” and he said it was my choice. (Mallapareddi et al., 2017)  What is AS? What is WW. (Loeb et al., 2018)  My wife is very supportive in the Watchful Waiting, which is extremely helpful, she’s not pressuring me to have it removed. (Kazer et al., 2011) |
| Regaining agency and driving resilience | They are going to watch me closely, on a structured program | …comfortable with the idea that they were going to watch me closely enough and with enough skill that if this began to be more aggressive or turn into something [else], that they would recognize it and then it would be time to get involved in [active treatment]. (Volk et al., 2014)  I think it gives me some comfort in being on active surveillance. If I was just drifting along and not thinking there was. . . some other possible alternative to the radical prostatectomy then I would yeah it just makes me feel better… (Oliffe et al., 2009)  …as it started to go on, and they started to explain to me and as time went on, and then more discussions with different doctors, I realised that if I’m monitored properly, then I can live as long as I would live anyway. (Eymech et al., 2022)  I really like the fact that [the hospital] was kind of putting me in a program. (Volk et al., 2014)  We’re going to deal with it in that I’m gonna have another biopsy this fall because it’ll be a year. That’s the way the active surveillance is set up... (Mader et al., 2017)  At times, not daily, at times, you know, there’s things you kind of have to put on the shelf, and, as they say in the movies, I’ll think about it tomorrow. (Davison et al., 2009) |
|  | These things validate my choice |  |
|  | Trust in the treating team | …mine was entirely on the “recommendation of my physician.” Uh, I trusted him. (Mallapareddi et al., 2017)  I had “confidence in the physician” that I selected. He’s got really good track record … (Mallapareddi et al., 2017)  I said, we are not going to worry about this because the doctor told us this was at the low-grade … (Mallapareddi et al., 2017)  I’d googled him. … endless qualifications, he travels overseas to conferences. (O'Callaghan et al., 2014)  Somebody you can “trust,” somebody that you know. Somebody that knows your medical history. (Mallapareddi et al., 2017)  I just listen to what the doctor said. Everybody swore by him. He’s got great reviews and I trusted what he said. (Loeb et al., 2018)  I like Dr. [name] I liked him, he’s a nice man, I guess some doctors you can like them and you trust them, some doctors you don’t know. (Mroz et al., 2013)  A big part of you making that decision is being “able to trust … doctor. (Mallapareddi et al., 2017)  He (doctor) went straight for active surveillance. It didn’t make any sense going around looking for other opinions really . . . I was quite happy with the deal sort of set up here and I wasn’t going to be bothered running around looking for alternatives. (Mroz et al., 2013)  I have confidence in him (doctor) and, and I felt confident with his advice and will continue to do so, I mean I think he’s fairly experienced in this. I’ve been fortunate in somebody like Dr. [name] who is pretty objective and he, he says, you know, “carry on,” um, and I’m able to live my life and so that I, um, I do that and in a sense when I have to really become serious about this, if I have to become serious I’ll deal with it then. (Mroz et al., 2013)  I put a lot of faith in them. I have a lot of trust in them. They’re very good at what they’re doing. (Mader et al., 2017)  I would always ask something like, ‘Do you feel like this is something that needs to be done now or do I need to take care of this now?’ and he usually said, ‘No, I don't think so.’ That to me was a pretty definitive statement … the last time, it was, ‘Well the numbers have changed, and they're going up’ … so, to me, it was not a rubber stamp of ‘Yup, let's keep in the program,’ and I promised myself that the first time I felt that I didn't get that rubber stamp, I'd do something about it. (Berger et al., 2014)  Whatever [the doctor] says. I’ll go along with that. The more confidence you have in the doctor, the better you feel. (Kazer et al., 2011)  I really would wait for the physician to say, you know, it’s time to take it out, take the prostate out. (Mader et al., 2017) |
|  | Satisfactory information provision | Especially in the first six months. You want to know everything You want to become your own expert. (Donachie et al., 2020)  I did the initial flurry of research and realized how the risk is minimal. If things change I will probably do more research later on. (Loeb et al., 2018)  I have a great source of information. My doctor. (Loeb et al., 2018)  I’m getting all the information I need [from my doctor]. If something comes along, I’d certainly like to be aware of it. Something innovative. (Loeb et al., 2018) |
|  | Family and peer support | I talked about it a lot. I looked up a lot of information that first week. I had to 'ruminate'. It helped me to process [..] (Donachie et al., 2020)  My Son and Daughter also have done an awful lot of research on it as well, once they found out that I had it and they were just filling me with more information and they thought it was a wise decision… (Kazer et al., 2011)  I talk a lot with the blokes, having a beer and yap about it’ (O'Callaghan et al., 2014)  I spoke to 20+ guys different ages from their 30’s up to my age who have been diagnosed with PCa. I learned a lot from them as to what worked, what didn’t, what they were doing and the like. (Loeb et al., 2018) |
|  | Treating team recommendation consistency | … I felt happy and reassured when someone told me what I wanted to hear … “You don’t need to have the operation.” (Mallapareddi et al., 2017)  I had been doing some processing and it was reassuring to hear him say the same thing as the first doctor. (Mader et al., 2017) |
|  | Favourable results | Yeah as long as my markers don’t change, uh, I’ll keep listening to the advice of my urologist. (Mallapareddi et al., 2017)  So, it’s seems like we are doing the right thing, and it gives me a little more peace of mind each three months when I go back in, well, we’re on the right track here. (Mader et al., 2017)  As long as things remain the same or increase slowly, uh, I intend to go active, uh, surveillance as long as I can. (Mallapareddi et al., 2017) |
|  | What can I do to help myself?  Lifestyle changes. | But ya gotta have a good outlook. And I’ve got probably one of the most positive women that I’ve ever met in my life for a wife, which is a big help. (Mader et al., 2017)  We’re always told that we should eat better and exercise more and avoid this and that, but if there are studies that have a direct correlation between PCa, of course I’d want it. (Loeb et al., 2018)  Just tips, point me in the right direction. What should you do, what shouldn't..[..] Food, lifestyle, that stuff.” (Donachie et al., 2020)  It went up. I used my diet and close attention to do all sorts of things to bring it down. But it still went up. That disappointed me. (Donachie et al., 2020)  She packs my lunch in the morning and makes sure I have all the right stuff, and a lot of fruits and vegetables, and none of the sweets I loved having (laughing)...went on with the tea, only have minimal beef from the beef industry and a lot of fruits and vegetables, and trying to get an alkaline diet rather than an acidic diet. So that’s what I tried to do to counteract this. (Mader et al., 2017)  And then the exercising was also on a website, I mean, exercising, I now joined a gym and I go to the gym two or three times a week and work out for about an hour. (Kazer et al., 2011)  . . . I’m not naïve enough to believe that I’m going to reverse things but I just sort of feel that maybe I can delay things a bit. (Oliffe et al., 2009)  I immediately changed my diet... we had greatly reduced the consumption of red meat and other fatty foods and our diet was pretty much chicken, fish, pasta, salads and then when I was diagnosed with prostate cancer and getting information off the web, they were suggesting more soy type products, tomato type products - tomato sauce, three times a week, sometimes four times a week and I’ve added shitake mushrooms to it, which supposedly on the web, it’s indicated that it’s a cancer fighter. (Kazer et al., 2011)  it was also suggested that I meet with one of their Directors in the nutrition department and many of the things that I follow have been based upon his recommendation as far as Vitamins, Supplements, foods to avoid and, hopefully, this is information that others would find helpful. [I avoid] fatty food. Most of the food is prepared with olive oil because of the Omega 3 makeup of the oil. I completely eliminated all eggs, butter and I haven’t had that in just many, many years…exercising, changing the diet, getting away from the fat free diets, more vegetables and fruits and so, I, it’s not all that complicated. (Kazer et al., 2011)  The things that are found now in my prostate are much better than 6 years ago. I don’t think anybody really knows why. I basically decided to change my diet considerably. (Loeb et al., 2018)  Well, those are antioxidants. I’m taking now Vitamin C, E, and I’m using garlic, which has been very beneficial in the intestine. And I’ve been taking beta carotene…nutritionally it’s probably quite beneficial,…in building up your body’s ability to combat this, particularly at the cellular level.…I think it can greatly deter its growth. (Bailey et al., 2004)  Take anything whether it’s proven or not . . . if there is a hint that it will slow the growth or reduce cancer in general. (Oliffe et al., 2009)  But ya gotta have a good outlook. And I’ve got probably one of the most positive women that I’ve ever met in my life for a wife, which is a big help. (Mader et al., 2017)  You know she’s kind of into a lot more healthy foods than I am. She always jokes about how it’s a work in progress to change my eating habits or whatever. I try to go along but don’t make it too dramatic…over time she’s kind of slowly changed things . . . but this situation [prostate cancer] has caused us to hasten the process a little bit more. (Oliffe et al., 2009)  . . . My wife is very aware of the dietary relationship with prostate cancer and so I eat a lot of vegetables, I eat a lot of fruit and all those things that I’m supposed to do, so salmon and all that stuff. (Oliffe et al., 2009)  They all haven’t got a supportive wife; without her, it wouldn’t have been such an experience for me where I felt confident about going forward. I think men who are on their own will find it much more difficult than maybe men who are married. (Eymech et al., 2022) |
